# Multi-informant reports of mental health in Swedish-born children of immigrants and children born to non-immigrants – the SESBiC-study

**DOI:** 10.1186/1471-2431-14-95

**Published:** 2014-04-08

**Authors:** Linda deKeyser, Carl Göran Svedin, Sara Agnafors, Marie Bladh, Gunilla Sydsjö

**Affiliations:** 1 Division of Obstetrics and Gynecology, Linköping University, SE-581 85 Linköping, Sweden; 2 Division of Child and Adolescent Psychiatry, Linköping University, SE-581 85 Linköping, Sweden

**Keywords:** Children of immigrants, Mental health, Multi-informant, Second-generation, SESBiC-study

## Abstract

**Background:**

The European literature on mental health of the children of immigrants is limited. Therefore this study aims to investigate gender-specific mental health reported by teachers, parents and the children themselves in 12-year old children of immigrants and non-immigrants and also to study the level of agreement between the different informants.

**Methods:**

This cross-sectional study is a part of the longitudinal South East Sweden Birth Cohort-study (the SESBiC-study) on children’s health. All children born in town in the south of Sweden 1995-1996 were invited to take part. The mothers of 1723 children (88%) consented. In this part 87 Swedish-born 12-year old children of immigrants and 687 12-year old children of non-immigrants were investigated regarding gender-specific differences in mental health as reported by teachers (Teacher-report form), parents (Child behavior checklist), and children (Strengths and Difficulties Questionnaire) and the agreement reached between the informants.

**Results:**

Parental immigrant status was not associated with mental health in any of the groups, but living arrangements and parental educational level were mainly found to have an effect on the health status of boys (TRF-Internalizing β = .77 95% CI = .02-1.52; TRF-Externalizing.β = 2.31 95% CI = .63-3.99; TRF-Total β = 6.22 95% CI = 2.27-10.18) The agreement between different informants was generally low, except for externalizing problems among boys (Boys of immigrant parents: Parent and teacher correlation ρ = .422 and Child teacher correlation ρ = .524, p-value < .05, respectively). The correlation between teachers and parents were lower in the index group compared to the reference group. In the index group, the correlations between teacher’s and children’s assessments were fairly high for boys but not for girls (ρ _Total_ = .400, ρ _Internalizing_ = .240 and ρ _Externalizing_ = .524, p-value < .05 for Total and Externalizing).

**Conclusion:**

This study confirms previous findings that the mental health of children of immigrants is similar to that of children of non-immigrants. We found that family factors have a greater impact on the reported mental health than immigrant status does. This might be of clinical importance for healthcare workers to recognize when investigating and treating children from other cultures.

## Background

The immigration to and within Europe have increased during the recent years. In 2012 there were 33.0 million people born outside a country of EU and there were an additional 17.2 million persons born in another country than the country of residence [1]. As a consequence we have a growing number of children born of immigrant parents and the number of children with an immigrant background continuous to rise in many countries but vary a lot from 39% in Switzerland to 17% in France [2] and in Sweden today 29.3% of all newborns [3] and 14% of adolescents in junior high school [4] are second-generation immigrants. This means that the wellbeing of such a large proportion of this young population is of great concern and of national interest.

The European literature on mental health in children of immigrants (referred to as the “second generation”), a fast growing group in our society, is very limited. It is therefore difficult to know if the mental health needs of these children might be different in some way compared to non-immigrant children. In recent reviews on mental health in migrants (of different generations) have emphasized that the results within this field of research are inconclusive and that it is hard to draw any general conclusions partly because of the variance in the definitions of the immigrant groups being studied [5-8]. In the latest review conducted on 36 studies on immigrant children [9] several studies showed that there were either no difference between first and second generation immigrant children or that first generation immigrant did worse concerning emotional or behavioural problems. Moreover, in many cases non-immigrant children reported more or as much behavioural problems as immigrant children, depending on who was the informant. They also pointed out several major influence factors in migrant children’s mental health, such as a low socio-economic status, a Non-European origin, an uncertain cultural identity of the parents, maternal harsh parenting or inadequate parental occupation, a minority status, the younger age, gender effects or a specific culture declaration in diseases.

In studies of well-defined groups, for example consisting only of the second generation and age relevant samples, the behaviour problems observed have been found to be similar to those in children of non-immigrants according to self- and parent-reports [10-16]. In studies where not all, but the majority (about 80%) were children of immigrants, a higher incidence of emotional and behaviour problems were found in children of immigrants compared to children of non-immigrants according to self-reports [17], parent-reports [18] and teacher-reports [16], except for a study by Crijnen, Bengi-Arslan & Verhulst (2000) where similar incidence was found according to teacher-reports [19]. The majority of the studies covered children of widely different age, making it difficult to draw sound conclusions since mental health varies considerably during childhood [20].

Findings on the emotional and behavioral problems among children fluctuate greatly depending on the informant used. The correlation between parent-, teacher- and self-reports using the Child behavior checklist (CBCL), the Teacher report form (TRF) and the youth self-report (YSR) respectively is modest, between .22-.28 and only a little bit higher between different informants using the Strengths and difficulties questionnaire (the SDQ), with correlations varying between .28 and .41 [21,22]. In the review by Belhadj Kouider et al. [9] only 8 of the 36 studies used two informants as the source for health and behavior information and the most common (five studies) was a combination of parent and teachers reports [9]. The children’s own views of their mental health are of great importance in order to reflect the strengths and difficulties that they themselves experience. Additionally, while parents have knowledge and perspectives on children’s functioning in the home environment, the teachers may have knowledge and perspectives on children’s functioning in different socially structured situations, learning situations and personal interactions with different people that leads to evaluations that differ from those of parents. Therefore, multi-informants are an important supplement to the parent-reports in understanding the children’s mental health. Teacher-reports on the children of immigrants are especially important since the parent’s way of interpreting - and their tolerance towards - certain types of behaviour might vary more widely than those of the teachers due to cultural differences [16,23,24].

The need for multi-informant, age- and gender-specific studies of mental health in a second generation group of children with immigrant and refugee parents led us to formulate the following goals: to investigate gender-specific mental health i.e. emotional and behavior problems reported by teachers, parents and the children themselves in 12-year old children of immigrants compared to children to non-immigrant parents and, in addition, to study the level of agreement between the different informants.

## Method

This cross-sectional study is a part of the longitudinal South East Sweden Birth Cohort-study (the SESBiC-study) on children’s health. The SESBiC-study’s primary objective is to study risk and resilience in children from early childhood until 12 years of age, but also to identify psychosocially burdened families and to identify risk factors for children’s mental development. All children born in the catchment areas of Hässleholm and Western Blekinge in the south of Sweden between May 1st 1995 and December 31st 1996 were invited to take part. The mothers of 1723 children (88%) consented. Follow-ups at age three, five and a half, and 12 have been performed and the results have been reported by Agnafors, Sydsjö, Dekeyser & Svedin (2012) [25], Dekeyser, Svedin, Agnafors & Sydsjö (2011) [12] Höök, Cederblad & Berg (2006) [26]. At the 12 year follow-up, 2 children had died, 11 had moved from Sweden and 24 were learning disabled. They were therefore excluded from the study reducing the original 1723 in the baseline study to 1686 in this study. Of these 1686, 1178 children, 923 parents and 983 teachers participated in the first phase.

For the purpose of this study, the number of children included was reduced to 774 (46% of the original 1686), using the criterion that all three informants must have completed the questionnaires for a child to be included in the study - 376 boys and 398 girls. Among those included in the study, 87 (11%) were Swedish-born children of immigrants, 51 with one immigrant parent (27 were mothers and 24 were fathers) and 36 with two immigrant parents. Of the immigrant parents, 21% were born in Nordic countries, 19% in Former Yugoslavia, 25% in Europe (excluding Former Yugoslavia) and 35% outside of Europe.

### Procedure at the 12 year follow-up

Home addresses were obtained from the Swedish Tax Office. Parents (i.e. legal guardians) received written information about the study as well as a consent form to sign in order to allow the child to participate in the study. The children received a simplified information letter. Research assistants met with the children in groups of 5–20 during school hours. All questions were read out loud by the research assistant and the children filled out the questionnaires without talking to each other. Children who no longer lived in the area or were not in school that day were scheduled for a home visit or a meeting at their new school. The individual child was given the same oral information and the research assistant was present in the room during the entire time when the child filled out the questionnaire. In a few cases, on the participant’s or parent’s initiative, the questionnaires were sent by mail to their home address. In addition, questionnaires for the parents were sent to the home address, which the mother and father were asked to fill out separately and return. In this paper we used the custodial parent’s questionnaire, when two custodial parents in the family participated the mother’s questionnaire was used since she had done this previously in earlier parts of the longitudinal study.

The families informed the research team about contact information of the teachers, thereafter the questionnaires were sent to the teacher’s work-address.

### Child and immigrant variables

All children who had at least one immigrant parent were considered to be Swedish-born children of immigrants and constituted the index group. Children whose both parents were born in Sweden were considered children of non-immigrants and formed the reference group. Information concerning parents’ country of origin, maternal life stress score (accumulation of social-, medical- and psychological stress factors) and the level of acculturation in the family i.e. if one or two parents were immigrants, if mother had lived at least five years in Sweden when the child was born, if Swedish was spoken at home, at least part of the time, if the country of origin was a Nordic or European country rather than a country outside of Europe was gathered at the time of the baseline study when the children were three months old. Information about emotional and behavioral problems among the children at the age of 3 was gathered at the 3-year follow up. Information about living arrangements, (i.e. whether the children were living with both parents or not) was collected from the children’s questionnaires at the 12-year follow up. Information about the parent’s educational level was collected from the parent’s questionnaires at the 12-year follow up. The children were thereafter grouped into higher education (>12 years of schooling) or lower education (<12 years of schooling) based on the parent with the highest education.

### Measures

The Strengths and Difficulties Questionnaire (SDQ) is a screening instrument that has good reliability and validity in different populations [27] both in industrialized and less developed countries worldwide [28]. It consists of 25 items divided between four problem subscales (*emotional-, conduct-, hyperactivity-* and *peer problems*) and one strengths subscale (*prosocial behavior*). The sum of the four problem subscales generates a *total difficulty score*. It was originally used for children aged 4–16 years and later also for 17–19 year olds [29]. In this study the self-report version was used and Cronbach’s alpha for the total scale was .66 (.67 for the reference group and .55 for the index group). The Child Behavior Check List (CBCL) and the teacher’s report form (TRF) are screening instruments of child behavior problems, designed for parents and teachers respectively. These screenings instruments, which have been translated into more than 60 languages, are being used worldwide and have shown good validity and reliability [30]. The 113 items, scored between 0 and 2, generate eight subscales: withdrawn, somatic complaints, anxious/depressed, social problems, thought problems, attention problems, delinquent behavior and aggressive behavior [30,31]. Those in turn generate the internalizing, externalizing and total problems scales. In this study the CBCL/4-18 and the TRF/5-18 were used [31]. A recent evaluation of the SDQ reported a significant correlation of .76 (total score) with the scales in the CBCL and TRF respectively - of the emotional symptoms in the SDQ with internalizing problems in the CBCL and TRF and of the conduct problems in the SDQ with externalizing problems in the CBCL and TRF [32].

### Dropout rate and analysis

The total dropout rate was 54%; 65% in the index group compared to 52% in the reference group (p < .001). Boys had a dropout rate of 58% compared to 50% among girls (p = .004). Children whose mothers had a high life stress score had a dropout rate of 72% compared to 53% among children whose mothers did not have a high life stress score (p < .001). However, no difference in mother’s life stress score was found within groups. Regarding earlier emotional and behavioral problems among the children at the age of 3, no significant difference was found. In the index group there was no difference in the dropout regarding gender, acculturation level or earlier emotional and behavior problems among the children at the age of 3.

### Data analysis

All analysis was performed using SPSS version 19.0. Statistical significance was defined as (two-sided) p < .05. Differences in means were analyzed using independent sample t-tests for significant group differences. To be able to compare the continuous scores on mental health by different informants a multivariate analysis (ANOVA) was performed for each of the psychometric measurements (TRF, CBCL, and SDQ) with immigrant status, living arrangement and parental education as independent variables. A sensitivity analysis of the multivariate analysis was performed to validate the results. The group of children of immigrants was divided into two groups, children of immigrants from Europe, excluding former Yugoslavia, and children or immigrants from outside Europe and former Yugoslavia. The inter-correlation of the three informants’ scores was calculated using Pearson correlations coefficient. For the dropout analysis Pearson’s chi-square test was used.

### Ethical considerations

The study was approved by the Ethics committee at the University of Lund in 1994 and 1998 and by the Regional Ethical Review Board in Linköping, 2007.

## Results

Demographic data on the parents in the two groups are present in Table 1. Prevalence numbers on mental health, measured by teachers (TRF), parents (CBCL) and self-reports by children (SDQ) on externalizing problems, internalizing problems and total problems for both boys and girls in the two groups are presented in Table 2.

**Table 1:** Socio-demographic background characteristics, Pearson’s chi-square unless otherwise indicated

	**Children of non- immigrant parents**		**Children of immigrant parents**		
	**n**	**%**	**n**	**%**	**p-value**
**Age when giving birth**					0.017
≤20	13	1.9	6	6.9	
21-37	653	95.1	78	89.7	
38-43	21	3.1	3	3.4	
**Maternal educational level**					0.719
Elementary	42	6.4	7	8.8	
High school	351	53.5	41	51.2	
University	263	40.1	32	40.0	
**Paternal educational level**					0.211
Elementary	66	13.8	13	22.4	
High school	288	60.1	32	55.2	
University	125	26.1	13	22.4	
**Maternal employment**					0.011*
Employed	641	99.2	76	95.0	
Not employed	5	0.8	4	5.0	
**Paternal employment**					0.011*
Employed	475	99.6	56	94.9	
Not employed	2	0.4	3	5.1	

*Fisher’s exact test due to few observations in two cells.

**Table 2:** **Prevalence numbers in the index**^**a**^**- and reference**^**b **^**group, with corresponding p-value, regarding mental health in reports by teachers (TRF), parents (CBCL) and children (SDQ)**

	**Immigrant status**	**Boys**			**Girls**		
		**n**	**%**	**Sig. (2-tailed)**	**n**	**Mean**	**Sig. (2-tailed)**
**Teacher (TRF)**							
Internalizing <10^th^ percentile	Index group^a^	7	13.2	,292	7	20.6	,055
	Reference group^b^	28	8.7		36	9.9	
Externalizing >90^th^ percentile	Index group^a^	14	26.4	,025	0	0.0	,610*
	Reference group^b^	46	14.2		11	3.0	
Total >90^th^ percentile	Index group^a^	8	15.1	,822	0	0.0	1,000*
	Reference group^b^	45	13.9		8	2.2	
**Parent (CBCL)**							
Internalizing <10^th^ percentile	Index group^a^	5	9.4	,380	7	20.6	,022
	Reference group^b^	20	6.2		31	8.5	
Externalizing >90^th^ percentile	Index group^a^	10	18.9	,151	2	5.9	1,000*
	Reference group^b^	38	11.8		27	7.4	
Total >90^th^ percentile	Index group^a^	9	17.0	,225	4	11.8	,308*
	Reference group^b^	36	11.1		26	7.1	
**Children (SDQ)**							
Internalizing <10^th^ percentile	Index group^a^	1	1.9	,488*	1	2.9	,123*
	Reference group^b^	17	5.3		42	11.5	
Externalizing >90^th^ percentile	Index group^a^	6	11.3	,462	0	0.0	,247*
	Reference group^b^	49	15.2		24	6.6	
Total >90^th^ percentile	Index group^a^	5	9.4	,809	1	2.9	,561*
	Reference group^b^	34	10.5		19	5.2	

*= Fisher’s exact test due to few number of observations in a cell.

^a^= Children of immigrants.

^b^= Children of non-immigrant parents.

In general, both teachers and parents reported more symptoms and behavioral problems among boys and girls in the index group compared to boys and girls in the reference group, but the only statistically significant difference was found in parent’s reports of internalizing problems in girls where more problems were found in the index group compared to the reference group (p = .026), Table 3. Although when living arrangements and parental educational level were adjusted for, those differences disappeared (Table 4). In the self-reports, the boys and girls in the index group generally reported symptoms and behavioral problems at the same or lower rate compared to the reference group and statistically significant differences were found among girls in the index group where they reported fewer emotional (p = .040) problems as well as total problems (p = .020) compared to girls in the reference group, Table 3. Once again those differences disappeared when living arrangements and parental educational level were adjusted for, Table 4.

**Table 3:** **Differences in mean scores in the index**^**a**^**- and reference**^**b **^**group, with corresponding p-value, regarding mental health in reports by teachers (TRF), parents (CBCL) and children (SDQ)**

	**Immigrant status**	**Boys**				**Girls**			
					**t-test of equality of means**				**t-test of equality of means**
		**n**	**Mean**	**SD**	**Sig. (2-tailed)**	**n**	**Mean**	**SD**	**Sig. (2-tailed)**
**Teacher (TRF)**									
Internalizing	Index group^a^	53	1.96	2.78	.878	34	2.68	3.05	.171
	Reference group^b^	323	1.90	3.02		364	1.91	3.09	
Externalizing	Index group^a^	53	4.87	6.53	.475	34	0.94	1.37	.311
	Reference group^b^	323	4.17	6.77		364	1.23	2.94	
Total	Index group^a^	53	15.00	15.43	.388	34	5.62	5.05	.802
	Reference group^b^	323	13.00	15.97		364	5.37	8.15	
**Parent (CBCL)**									
Internalizing	Index group^a^	53	4.45	4.06	.700	34	7.00	5.09	.026
	Reference group^b^	323	4.22	4.53		364	4.89	5.03	
Externalizing	Index group^a^	53	6.34	5.84	.287	34	4.35	5.04	.685
	Reference group^b^	323	5.39	6.76		364	3.99	4.63	
Total	Index group^a^	53	15.96	11.73	.389	34	15.97	12.68	.112
	Reference group^b^	323	14.39	15.18		364	12.30	11.63	
**Children (SDQ)**									
Internalizing	Index group^a^	53	1.83	1.54	.545	34	2.21	1.47	.040
	Reference group^b^	323	1.97	1.78		364	2.78	1.97	
Externalizing	Index group^a^	53	1.72	1.47	.786	34	1.47	1.16	.590
	Reference group^b^	323	1.78	1.58		364	1.36	1.21	
Total	Index group^a^	53	11.06	5.84	.752	34	8.44	4.61	.020
	Reference group^b^	323	11.33	6.27		364	10.48	5.60	

^a^= Children of immigrants.

^b^= Children of non-immigrant parents.

**Table 4:** Multivariate ANOVA coefficients and 95% CI regarding mental health in reports by teachers (TRF), parents (CBCL) and children (SDQ), reported by gender and informant

	**Internalizing**		**Externalizing**		**Total**	
	**B (95% CI)**	**p-value**	**B (95% CI)**	**p-value**	**B (95% CI)**	**p-value**
**Teacher (TRF)**						
**Boys**						
Immigrant status^a^	0.54(−0.82-0.93)	.903	0.60 (−1.36-2.56)	.548	1.75 (−2.87-6.37)	.456
Living arrangement^b^	0.77(0.02-1.52)	.043	2.31(0.63-3.99)	.007	6.22(2.27-10.18)	.002
Parental educational level^c^	0.06(−0.55-0.67)	.846	1.03(−0.33-2.39)	.138	2.14(−1.07-5.33)	.190
**Girls**						
Immigrant status^a^	0.43(−0.74-1.61)	.467	−0.23(−1.31-0.85)	.674	0.23(−2.79-3.24)	.882
Living arrangements^b^	0.25(−0.49-0.98)	.509	0.54(−0.14-1.22)	.118	1.42(−0.46-3.30)	.138
Parental educational level^c^	0.18(−0.44-0.80)	.565	0.16(−0.42-0.73)	.594	0.87(−0.72-2.46)	.282
**Parents (CBCL)**						
**Boys**						
Immigrant status^a^	0.60(−0.59-1.79)	.324	1.36(−0.40-3.11)	.129	2.91(−0.95-6.76)	.139
Living arrangements^b^	1.43(0.47-2.39)	.004	1.34(−0.08-2.75)	.064	4.45(1.33-7.56)	.005
Parental educational level^c^	0.40(−0.41-1.22)	.328	1.44(0.25-2.64)	.018	2.41(−0.23-5.04)	.073
**Girls**						
Immigrant status^a^	0.92(−0.73-2.57)	.273	0.61(−0.88-2.09)	.422	2.44(−1.36-6.24)	.207
Living arrangements^b^	0.61(−0.44-1.65)	.255	1.31(0.37-2.24)	.006	3.17(0.76-5.58)	.010
Parental educational level^c^	0.64(−0.28-1.56)	.171	0.42(−0.41-1.24)	.324	2.04(−0.08-4.17)	.059
**Children (SDQ)**						
**Boys**						
Immigrant status^a^	0.09(−0.38-0.56)	.715	0.13(−0.29-0.55)	.541	0.11(−1.57-1.78)	.901
Living arrangements^b^	0.28(−0.10-0.66)	.152	0.14(−0.20-0.48)	.411	0.31(−1.05-1.67)	.656
Parental educational level^c^	−0.03(−0.35-0.30)	.877	0.031(−0.26-0.32)	.833	0.04(−1.11-1.19)	.943
**Girls**						
Immigrant status^a^	−0.33(−1.00-0.33)	.322	−0.08(−0.49-0.32)	.683	−1.50(−3.36-0.37)	.115
Living arrangements^b^	0.11(−0.31-0.53)	.593	0.27(0.01-0.53)	.039	1.42(0.24-2.60)	.019
Parental educational level^c^	0.45(0.08-0.82)	.017	0.10(−0.13-0.33)	.390	1.28(0.24-2.32)	.016

^a^0 = Children of immigrants and 1 = Children of non-immigrants = reference.

^b^0 = not living with both parents (includes children with foster parents) and 1 = living with both parents = reference.

^c^0 = higher education (university or college degree) = reference and 1 = lower education (not university or college degree).

In the multivariate analysis where adjustments were made for immigrant status, parental educational level and living arrangements it was found that if the child lived with both parents or not became a significant variable for mental health. In reports by teachers, boys who were not living with both parents had more internalizing problems (p = .043), externalizing problems (p = .007) and total problems (p = .002) compared to boys who were living with both parents, Table 4. No differences were found among girls, Table 4. In reports by parents, boys who were not living with both parents had more internalizing problems (p = .004) and total problems (p = .005) compared to boys who were living with both parents. Furthermore, in reports by parents, girls who were not living with both parents had more externalizing problems (p = .006) and total problems (p = .010), Table 3. In reports from the children, no difference was found among boys. Girls who were not living with both parents had more externalizing problems (p = .039) and total problems (p = .019) compared to girls who were living with both parents, Table 4. Furthermore, parental educational level was associated with externalizing problems reported by parents, where boys whose parents had lower education had more problems (p = .018) compared to boys whose parents had higher education, Table 3. Moreover, parental education level was associated with internalizing problems reported by the children where girls whose parents had lower education had more internalizing problems (p = .017) and total problems (p = .016) compared to girls whose parents had higher education, Table 4. No statistically significant differences were detected between those who had one immigrant parent and those where both parents were immigrated on any of the psychometric scales (data not shown).

**Figure 1:**
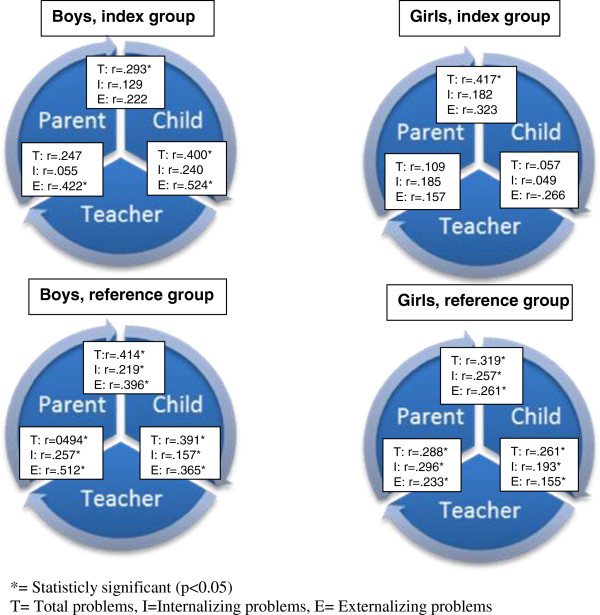
Correlation (r) between teacher, parent and child scores on TRF, CBCL and SDQ respectively by gender in the index group, i.e. preadolescents of immigrants, and the reference group, i.e. preadolescents of non-immigrants.

In Figure 1, correlations show that the agreement between different informants was generally low, except for externalizing problems among boys. The correlation between teachers and parents were lower in the index group compared to the reference group (Figure 1). In the index group, the correlations between teacher’s and children’s assessments were fairly high for boys but not for girls, Figure 1.

In order to verify the results, a sensitivity analysis of the multivariate analysis was performed. In the revised analyses the group of children of immigrants was divided into two groups, children of immigrants from Europe, excluding former Yugoslavia, and children or immigrants from outside Europe and former Yugoslavia. This change in grouping of children of immigrants did not change the results significantly (data not shown).

## Discussion

In this gender- and age-specific study, parent-, teacher- and self-reports of mental health in Swedish-born children of immigrants and non-immigrants were investigated together with the level of agreement between the three informants. The results can be summarized in the following three main findings.

First, these findings confirm what was previously found in the SESBiC-study [12] and other studies [10-16] – that the mental health in children of immigrants is very similar to that of children of non-immigrants according to teachers, parents and self-reports. This study and the study by Vollebergh et al., 2005 [24], found that parents reported more internalizing problems while the children themselves reported fewer internalizing problems in the daughters of immigrants compared to the daughters of non-immigrants, but in this study those differences disappeared when family factors such as living arrangement and parental education were adjusted for.

Second, in the multivariate analysis, immigrant status was not associated with mental health in any of the reports, but in line with other studies [33], parent’s educational level and living arrangements were. This indicates that family factors matter more than the parents’ immigrant status in determining mental health in children of immigrants at the age of 12 in Sweden.

This was also confirmed in the review by Belhadj and colleges (2013) were they concluded that factors influencing mental health status in children of immigrants were among others uncertain cultural identity and educational/occupational status of parents as well as harsh maternal parenting [9].

Third, the inter-informant agreement in this study was generally low and also in line with results of other studies [20,22]. The large discrepancies indicate the importance of good communication between the school and the family for a better joint understanding and overall view of the child’s behaviour in all social settings. It is especially important that professionals are aware of these discrepancies so they can be taken into account in planning interventions. In a review article by De Los Reyes & Kazdin (2005) higher agreement was found in externalizing problems compared to internalizing problems and that is in line with our results in boys, but not in girls [34]. This is probably because externalizing problems are easier to detect than internalizing problems especially in a school setting. The low correlation between the evaluations of teachers and parents found in the index group could indicate that the informants meet the children in different social settings and, perhaps, the children in the index group act more differently in school than at home compared to children in the reference group perhaps due to different values and social rules. It could also be explained by the knowledge and attitudes about what deviant behaviour is or due to bias in reports. In the event of future mental health problems, these differences may be of importance when developing treatment programs.

A limitation of this study is the fact that the sample size was too small to gain adequate statistical power for further analysis in subgroups based on parent’s country of origin or parent’s reasons for migrating. Therefore, the great variation of cultural background in our index group means that we cannot generalize our results for all subgroups of children of immigrants. Another limitation of the study is the high dropout rate that often burdens longitudinal studies. The study is a follow-up of a birth cohort over a 12-year period, and only those that agreed to participate in this follow-up at the age of 12 and have assessments from all three informants were included in the study. One explanation of the dropout in the index group could be that the parents had difficulty understanding Swedish, although they had lived in Sweden for at least twelve years, and thus felt it problematic to take part in a study with such comprehensive instruments as this study used. We also must acknowledge the risk that some of the participants might have had trouble to fully interpret the questions in the instruments and thus have had an influence on the answers. The higher dropout in the group where mothers reported a high life stress score could be due to a lower ability to engage in a study due to existing time and energy constraints and therefore dropout to a higher degree. When further analyzing the dropout in the index group specifically, no differences were found regarding gender, mother’s life stress score or earlier emotional and behavior problems among the children at the age of 3 between participants and dropouts.

An important strength of our study is that it is a part of a birth cohort and we were therefore able to use a well-defined index group children of immigrants only, i.e. all the children in the study were born in Sweden and none of them have experienced the migration process or been exposed to trauma during war and stress related to being a refuge. We find it important to separate the children of immigrants, i.e. the second-generation, from the first-generation since children from the second generation have better psychological adaptation than the first generation – findings from a study of five European countries [35]. Findings in Dutch studies suggest that child-, school/peer- and family factors and not migration factors are the strongest predictors for behaviour problems in the second-generation [36] and that is in line with what we found in this study.

## Conclusion

This study confirms previous findings that mental health in children of immigrants are generally equivalent to that of children of non-immigrants and that family factors like living arrangements and parental educational level have a greater impact on their mental health than their parent’s immigrant status. This might be of clinical importance for healthcare workers to recognize when investigating and treating children from other cultures. Lastly, teachers and parents disagree more regarding mental health in children of immigrants compared to children of non-immigrants and that might have an implication for the child regarding support and care both in school and in health care.

## Competing interests

The authors have no interest to declare.

## Authors’ contribution

C-G S, GS planned and designed the study. LDK and SA contributed to the acquisition of data. All authors were involved in the analyse process of the data and MB provided statistical support throughout the working process. LDK was primarily responsible for writing the paper. All authors were involved in the drafting and revising of the paper and approved the final version of the manuscript for submission.

## Pre-publication history

The pre-publication history for this paper can be accessed here:

http://www.biomedcentral.com/1471-2431/14/95/prepub
